# Egg Shell Membrane as an Alternative Vascular Patch for Arterial Angioplasty

**DOI:** 10.3389/fbioe.2022.843590

**Published:** 2022-03-18

**Authors:** Peng Sun, Shujie Yan, Liwei Zhang, Cong Zhang, Haoliang Wu, Shunbo Wei, Boao Xie, Xiaofeng Wang, Hualong Bai

**Affiliations:** ^1^ Department of Vascular and Endovascular Surgery, First Affiliated Hospital of Zhengzhou University, Zhengzhou, China; ^2^ Key Vascular Physiology and Applied Research Laboratory of Zhengzhou City, Zhengzhou, China; ^3^ National Center for International Research of Micro-Nano Molding Technology, Key Laboratory of Henan Province for Micro Molding Technology, Zhengzhou, China; ^4^ School of Mechanics Science and Safety Engineering, Zhengzhou University, Zhengzhou, China

**Keywords:** egg shell membrane, patch angioplasty, decellularization, heparin, neointimal hyperplasia

## Abstract

**Introduction:** The egg shell membrane (ESM) is always considered as waste, but recent studies have shown that it has the potential to yield rapid re-endothelialization *in vitro*. We hypothesized that ESM and heparin-conjugated ESM (HESM) can be used as arterial patch in a rat aortic angioplasty model.

**Method:** Sprague-Dawley rat (200 g) abdominal aortic patch angioplasty model was used. Decellularized rat thoracic aorta (TA) patch was used as the control; ESM patch was made of raw chicken egg; heparin-coated ESM (HESM) patch was made by using dopamine; anticoagulation properties were verified using platelet adhesion tests; the TA, ESM, and HESM patches were implanted to the rat aorta and harvested at day 14; and the samples were examined by immunohistochemistry and immunofluorescence.

**Result:** The ESM patch showed a similar healing process to the TA patch; the cells could migrate and infiltrate into both patches; there was a neointima with von Willebrand factor-positive endothelial cells; the endothelial cells acquired arterial identity with Ephrin-B2- and dll-4-positive cells; there were proliferating cell nuclear antigen (PCNA)-positive cells, and PCNA and alpha smooth muscle actin dual-positive cells in the neointima in both groups. Heparin was conjugated to the patch successfully and showed a strong anticoagulation property *in vitro*. HESM could decrease mural thrombus formation after rat aortic patch angioplasty.

**Conclusion:** The ESM is a natural scaffold that can be used as a vascular patch; it showed a similar healing process to decellularized TA patch; HESM showed anticoagulation property both *in vitro* and *in vivo*; and the ESM may be a promising vascular graft in the clinic.

## Highlights

Egg shell membrane can be used as a novel arterial vascular patch
Egg shell membrane can be surface modified and be used as a tissue engineered vascular graft.

## Introduction

Patch angioplasty is a commonly used technique to prohibit potential stenosis after vascular surgery, traditional patch materials like prosthetic Dacron and expanded polytetrafluoroethylene patches have contributed to the advancement of vascular surgery, but as foreign materials, they have the potential risk of infection and pseudoaneurysm formation (den Hoed and Veen, 1992; Eads and Ikonomidis, 2014). Autologous vein patch is also commonly used, but it is inclined to bulge after implantation because of high compliance (Esposito et al., 2021). So better or novel materials are needed in both the clinic and basic research, and with the rapid development of science and techniques in biomaterials science, natural materials have attracted more and more attention. Various new natural materials have been tested for their application as patches in clinics or basic researches, like the peritoneum–fascia patch (Sarac et al., 2005), small intestinal submucosal (SIS) graft (Magden et al., 2021), bovine and porcine pericardial patch (Bai et al., 2018a; Song et al., 2021), plant-derived patch (Bai et al., 2021a; Xie et al., 2021), fish swim bladder patch (Bai et al., 2021b), and biomimetic elastin fiber patch (Bai et al., 2021c). All these materials have some merits and drawbacks (Bai et al., 2021d).

The egg shell membrane (ESM) is always considered as waste, but recent studies have shown that it has exceptional properties. The structure and composition of the ESM has been reviewed in several researches (Balaz, 2014; Sah and Rath, 2016; Choi et al., 2021). Besides the wide application in materials and industry researches, the ESM has also been used in the treatment of pain in joint and connective tissue disorders (Ruff et al., 2009) and as an anti-inflammatory biomaterial (Benson et al., 2012). It can also accelerate burned skin healing (Maeda and Sasaki, 1982) and nerve regeneration (Farjah et al., 2013). Moreover, the ESM when cross-linked with gelatin–chitosan cryogels can be a skin substitute (Saha et al., 2021). These applications in different fields of the ESM have proved that it is a promising biomaterial in medicine. We recently showed that the ESM has the potential to yield rapid re-endothelialization *in vitro*. A small-diameter, double-layered ESM/thermoplastic polyurethane (TPU) vascular graft with a wavy structure was developed, and heparin was successfully coated on the ESM surface and showed anticoagulation properties (Yan et al., 2020). The reason we used TPU in this previous research was to enhance the strength and keep the tube structure of the ESM, but as a natural material, whether the ESM alone can be used as a vascular patch is still unknown. Since a lot of ESMs are being discarded every day, and if these could be recycled and used in vascular research or future clinical applications, then this trash could become potential useful biomaterials.

To mimic the human arterial patch angioplasty, we established a rat aortic patch angioplasty model (Bai et al., 2017a). We also showed that the patch coated with programmed death-1 (PD-1) inhibitor or heparin can decrease neointimal hyperplasia (Bai et al., 2020; Bai et al., 2021e). Heparin is an anticoagulant that inhibits acute thrombosis after vascular surgery. It also shows the function of inhibiting neointimal hyperplasia (Bai et al., 2020). Based on previous researches, we hypothesized that pure ESM can be used as a potential arterial patch and heparin-coated ESM (HESM) patch can decrease thrombus formation after implantation; we therefore tested our hypothesis in the rat aortic patch angioplasty model.

## Methods

The animal experiments were authorized by the First Affiliated Hospital of Zhengzhou University Animal Experiment Ethics Committee and Authority for Animal Protection. All animal experiments complied with the ARRIVE guidelines and were carried out in accordance with the United Kingdom's The Animals (Scientific Procedures) Act 1986 and its associated guidelines.

### Making Egg Shell Membrane Patch

**FIGURE 1:**
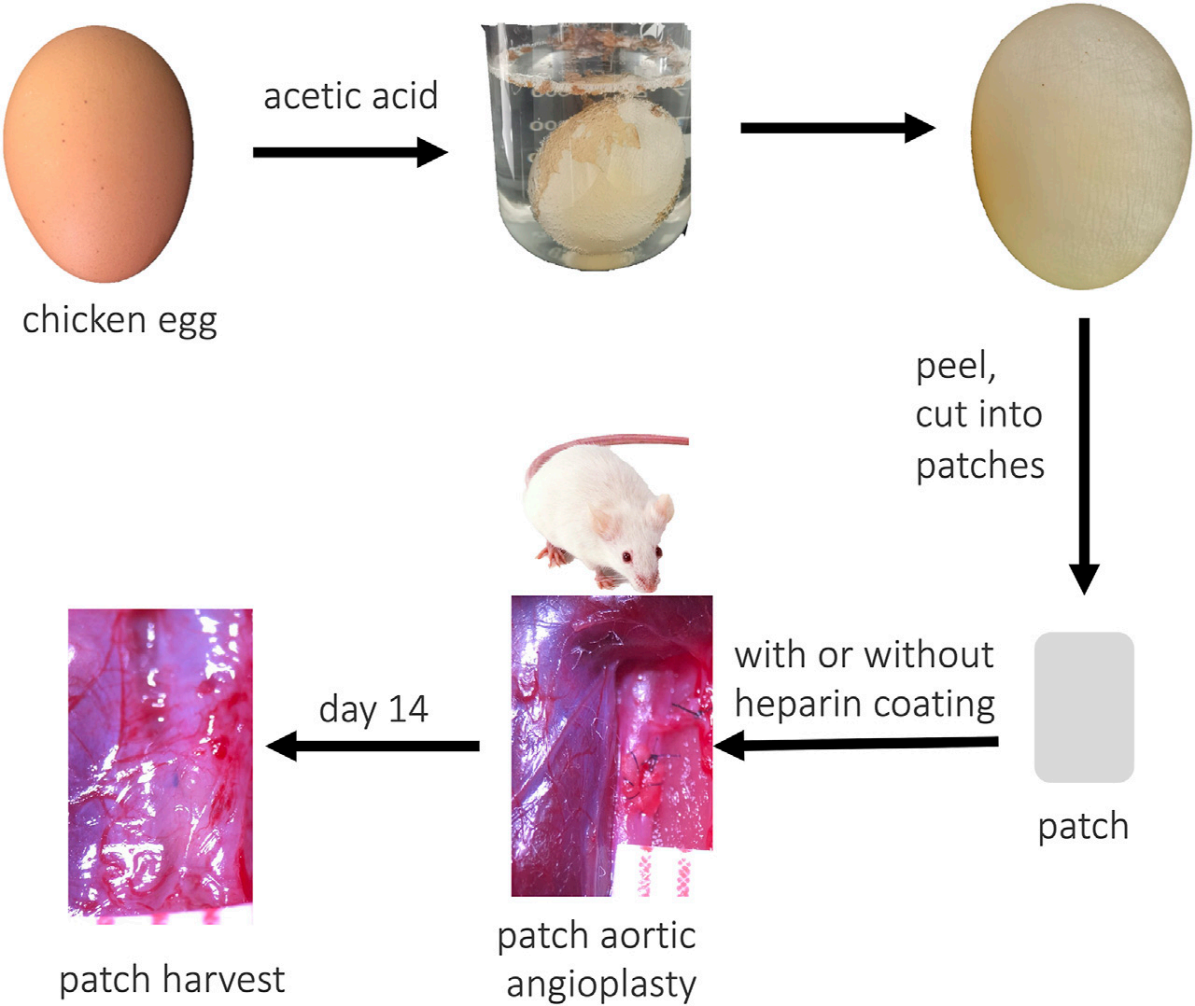
Illustration photograph showing the study design.

A raw chicken egg was washed and put into 1% acetic acid. After 24 h of soaking, the egg was washed using running water and the ESM peeled off and washed with distilled water. The ESM was then carefully cut into patches (3 × 1.5 mm) (Figure 1). The patches were examined by scanning electron microscopy (SEM).

### Heparin Covalent to Egg Shell Membrane

The ESM was treated with dopamine (DA) and heparin as we have previously described (Yan et al., 2020). First, a tris(hydroxymethyl) aminomethane solution was prepared (10 mM, pH 8.5) as the buffer to dissolve the dopamine. The heparin solution (1 mg/ml) was prepared using the abovementioned buffer. The ESMs were immersed into the heparin solution at 4°C for 12 h, which was enough time for the heparin to covalently bond. These samples were named HESM.

### Platelet Adhesion Test

Platelet adhesion assay was performed as we have previously described (Yan et al., 2020). The original ESM and HESM samples were used in the platelet adhesion tests. Single-donor human whole blood was purchased from Innovative Research. The blood was added into the centrifuge tube and followed by centrifuging at 2000 rpm for 10 min to obtain platelet-rich plasma (PRP). Then, 200 μl of PRP was added to each sample, and it was incubated at 37°C for 2 h. After incubation, the samples were rinsed three times with PBS and the absorbed platelets were fixed with 2.5 wt% glutaraldehyde at 4°C overnight. Then the samples were dehydrated with different concentrations of alcohol (50, 60, 70, 80, 90, and 100%) for 15 min each and sufficiently dried in a freeze dryer, then followed by platinum coating and imaging using SEM.

### *In Vitro* Cytocompatibility Assessment

Experiments were carried out as we have previously described (Yan et al., 2020). The human umbilical vein endothelial cells (HUVECs) were purchased from Lonza. They were used for cell viability testing to assess the feasibility of ESM and HESM. The culture was performed in a thermostatic incubator at 37°C in a 5% CO_2_ and 95% air environment. At confluence, HUVECs were dissociated with TrypLE Express enzyme (Gibco) to obtain suspended cells, which were centrifuged and resuspended in medium prior to cell seeding.

The HUVECs on the ESM and HESM patches were evaluated by phalloidin/4′,6-diamidino-2-phenylindole (DAPI) staining and the LIVE/DEAD assay. To further confirm the live and dead cells, fluorescence staining was performed on chosen samples using a LIVE/DEAD Viability/Cytotoxicity Kit (Life Technologies). Cell viability was determined after culturing for 7 days. A kit containing green fluorescent calcein-AM and red fluorescence ethidium homodimer-1 (EthD-1) was used to stain both live and dead cells simultaneously. The samples were immersed in a staining solution (300 μl/cm^2^) away from light for 20 min at 24°C and then imaged with a Nikon fluorescence microscope.

DAPI (Sigma-Aldrich) and phalloidin fluorescent staining (Biotium) were used for marking the cell nuclei and cytoskeleton f-actin molecules, respectively, to visualize the HUVECs morphology on the samples. The samples with HUVECs were washed three times, then fixed with 4% paraformaldehyde in PBS for 15 min on ice, followed by a PBS rinse, and then permeabilized with 0.5% Triton X-100 in PBS for 10 min. Next, red fluorescent phalloidin solution was used to stain the cell cytoskeleton for 20 min and blue fluorescent DAPI solution used to stain the cell nuclei for 5 min; these were carried out both away from light and at room temperature. The staining processes followed the manufacturer's recommendations. The images were then captured at day 7 using the same fluorescence microscope.

### Rat Thoracic Aorta Decellularization

The decellularization procedure was carried out as we have previously described (Bai et al., 2021e). Briefly, the thoracic aorta (TA) was harvested and incubated in 10 ml of sodium dodecyl sulfate buffer (1.8 mM sodium dodecyl sulfate, 1 M NaCl, and 25 mM EDTA in PBS) for 24 h and then rinsed with PBS; the decellularized TA was cut into patches (3 × 1.5 mm).

### Animal Model

Male Sprague-Dawley rats (aged 6–8 weeks) were used and anesthetized with an intraperitoneal injection of 10% chloral hydrate (Wu et al., 2021). Adequate anesthesia was confirmed by a lack of response to a toe and tail pinch, ointment was placed on the eyes to prevent dryness while the animals were under anesthesia, and the ventral abdomen hair was removed using a hair remover while wearing sterile gloves. For postoperative analgesia, buprenorphine was given at 0.1 mg/kg intramuscularly, no less than every 12 h for 24 h following the surgical procedures. The status of the animal was checked every day in the animal room, ensuring proper recovery from the perioperative period as well as adequate treatment of postsurgical pain. The aorta patch angioplasty model was performed as we have previously described (Bai et al., 2017b). The microsurgical procedures were performed aseptically using a dissecting microscope (Nikon, Japan). The TA, ESM, and HESM patches (approximately 3 × 1.5 mm^2^) were implanted to the infrarenal aorta of the rats using continuous 11-0 nylon sutures; the eggshell side of the ESM was facing the aortic lumen. The rats were sacrificed on postoperative day 14, and the patches were explanted for analysis. No immunosuppressive agents, antibiotics, or antiplatelet agents were administered at any time.

### Histology Staining

The rats were anesthetized with an intraperitoneal injection of 10% chloral hydrate, and the tissues were fixed with transcardial perfusion of PBS, followed by that of 10% formalin. The tissues were removed and fixed overnight in 10% formalin followed by a 24 h immersion in 70% alcohol. They were then embedded in paraffin and sectioned (4-μm thickness). The tissue sections were deparaffinized and stained using the hematoxylin and eosin (H&E) staining kit (Baso, Zhuhai, China) according to the manufacturer's recommendations. Neointimal thickness was the mean of the measurements taken from the edge of the surface to the edge of the patch in three independent areas.

### Immunohistochemistry

The sections were heated in a citric acid buffer (pH 6.0, Beyotime, Shanghai, China) at 100°C for 10 min for antigen retrieval. They were then treated with 0.3% hydrogen peroxide for 30 min and incubated overnight at 4°C with primary antibodies (Table 1). After overnight incubation, the sections were incubated with appropriate secondary antibodies (Table 1) for 1 h at room temperature and treated with the 3,3N-diaminobenzidine tetrahydrochloride horseradish peroxidase Color Development Kit (Beyotime, Shanghai, China) to detect the reaction products. Finally, the sections were counterstained with hematoxylin (Baso, Zhuhai, China). The positive cells were counted and expressed as n/mm^2^.

**TABLE 1:** Antibodies used in this experiment.

Antibodies	Vendor	Lot number	Concentration
Primary antibody			
CD34	Abcam	Ab81289	1:100
Nestin	Abcam	Ab11306	1:100
CD68	Abcam	Ab31360	1:100
Alpha smooth muscle cell (α-SMA)	Abcam	Ab5694	1:200
Ephrin-B2	ABclonal	A12961	1:100
dll-4	ABclonal	A12943	1:100
Cleaved caspase-3	Cell Signaling Technology	9661	1:50
PCNA	Abcam	Ab29	1:50
vWF	Abcam	Ab11713	1:100
IL-10	R&D	AF-401-NA	1:100
TNF-α	Abcam	Ab6671	1:50
Secondary antibody			
Goat anti-rabbit	Bioworld	BS12478	1:100
Goat anti-mouse	Bioworld	BS13278	1:100
488 Goat anti-Mouse	ABclonal	AS073	1:100
CY3 Goat anti-rabbit	ABclonal	AS007	1:100

### Immunofluorescence

Tissue sections were deparaffinized and then incubated with primary antibodies (Table 1) overnight at 4°C. The sections were incubated with secondary antibodies (Table 1) for 1 h at room temperature; subsequently, the sections were stained with the fluorescent dye DAPI (Solarbio, Beijing, China) to stain cellular nuclei. The positive cells were counted and expressed as n/mm^2^.

### Statistical Analyses

Data are expressed as mean ± standard errors of mean. Statistical significance for these analyses was determined using t tests (Prism 6; GraphPad Software, La Jolla, CA, United States). *p* values <0.05 were considered significant.

## Results

### Egg Shell Membrane Shared a Similar Healing Process Like Decellularized Rat Thoracic Aorta Patch

**FIGURE 2:**
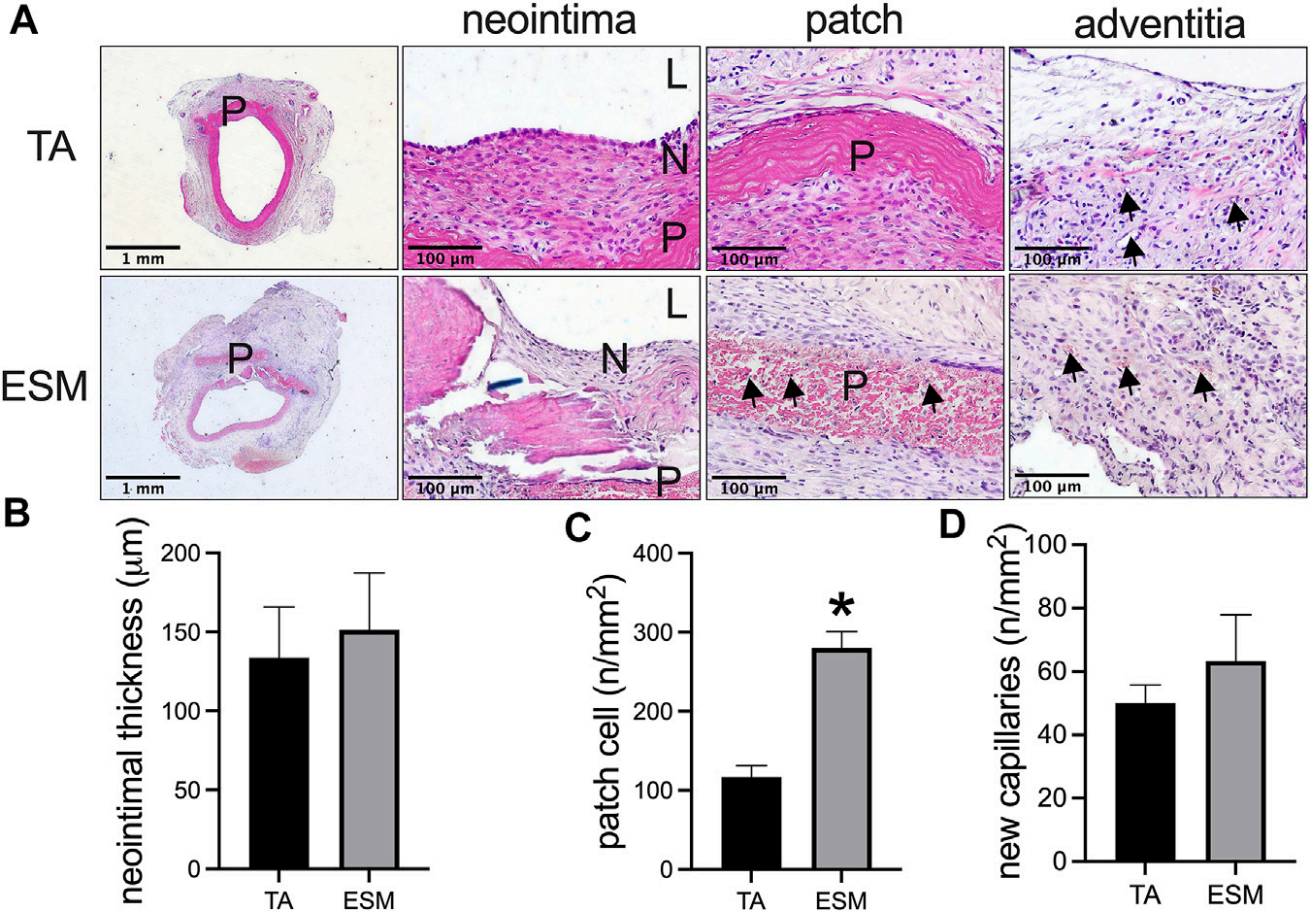
Comparison of the egg shell membrane (ESM) patch to the decellularized rat thoracic aorta patch harvested from the rat aortic angioplasty model at day 14. **(A)** Photographs of hematoxylin & eosin staining of the patches after angioplasty at day 14. High power photographs showing the neointima, cell infiltrated into the patch, and adventitia; scale bar, 1 mm or 100 μm; L, lumen; P, patch; N, neointima; black arrows show the cells infiltrated into the ESM patch or new capillaries in the adventitia, *n* = 3. **(B)** Bar graph showing the neointimal thickness in the angioplasty model at day 14, *p = 0.7322, *t*-test, *n* = 3. **(C)** Bar graph showing the number of cells infiltrated into the patch in the angioplasty model at day 14, *p = 0.0030, *t*-test, *n* = 3. **(D)** Bar graph showing the number of adventitial new capillaries in the angioplasty model at day 14, *p* = 0.4418, *t*-test, *n* = 3.

A chicken egg was immersed in 1% acetic acid solution; after 24 h of soaking, bubbles were observed from the egg shell, and the outside part of the egg shell dissolved; the egg became soft and smooth (Figure 1). Then, the ESM was cut into patches and implanted to the rat abdominal aorta, and decellularized rat TA patch was used as the control. After 14 days, the ESM was incorporated to the native aorta and capsuled by newly formed tissue (Figure 1). H&E staining showed neointima formed on the luminal side of the TA and ESM patches (Figure 2A), and the neointimal thickness was similar in both groups (Figure 2B). In the TA patch, there were few cells infiltrated into the TA patch, but there were more cells infiltrated into the ESM patch compared to the TA patch (Figures 2A,C). A thick adventitia was observed in both the groups, and there was no difference in the number of newly formed capillaries in both the patches (Figures 2A,D).

**FIGURE 3:**
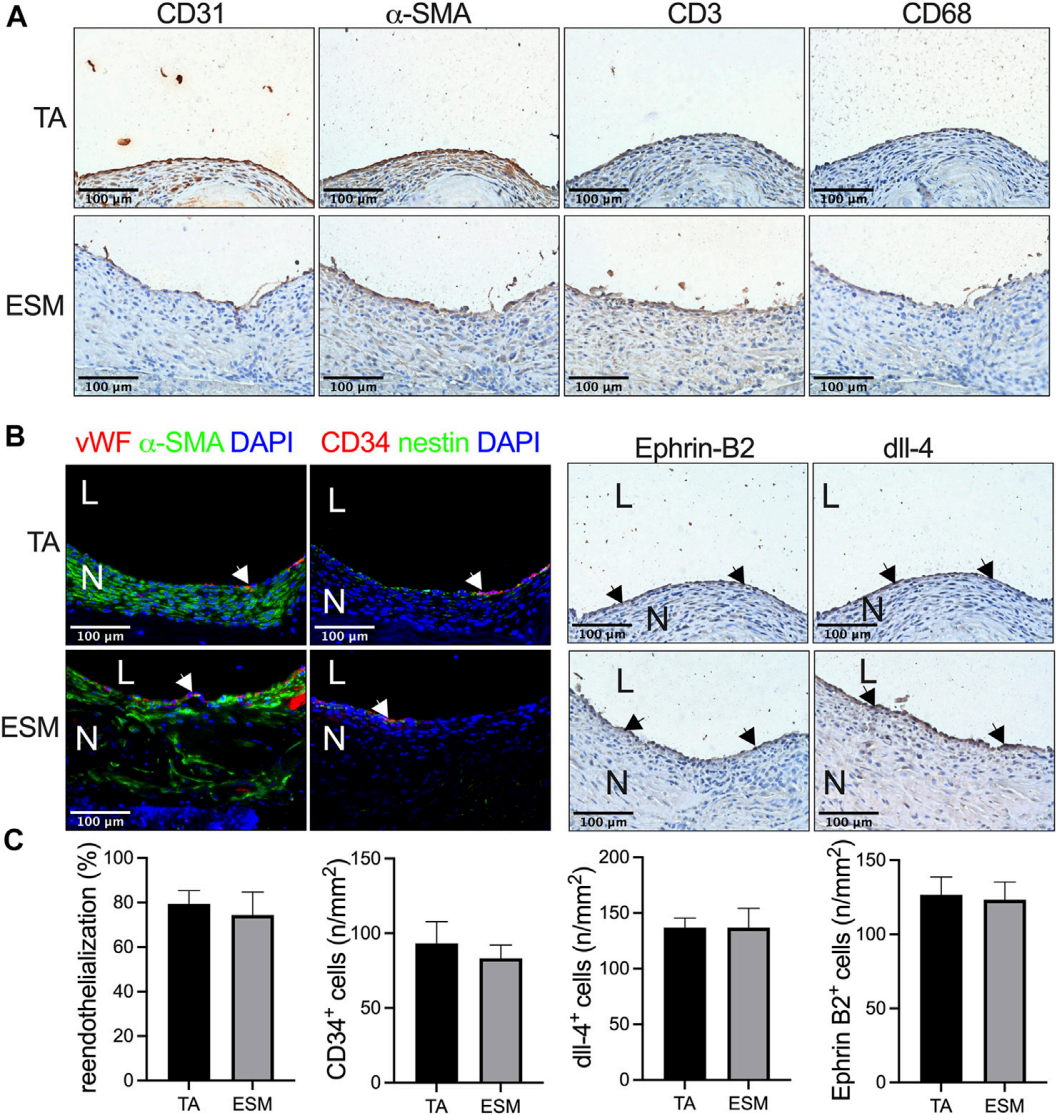
Neointimal cell composition after ESM patch angioplasty at day 14. **(A)** Photographs of immunohistochemistry stained for CD31, α-actin, CD3, and CD68 in the neointima in the TA and SEM patches; scale bar, 100 μm; *n* = 3. **(B)** Photographs of the immunofluorescence staining for von Willebrand factor (red), α-actin(green), and DAPI (blue); CD34 (red), nestin (green), and DAPI (blue); photograph of immunohistochemical staining for Ephrin-B2 and dll-4 of the neointima; scale bar, 100 μm; L, lumen; N, neointima; white or black arrows show the positive cell, *n* = 3. **(C)** Bar graphs showing re-endothelialization (*p* = 0.6985, *t*-test), CD34-positive cell numbers (*p* = 0.5879, *t*-test), dll-4-positive cell numbers (*p* = 0.8541, *t*-test), and Ephrin-B2-positive cell numbers (*p* > 0.9999, *t*-test) in the TA and ESM patch groups at day 14, *n* = 3.

We then examined the neointimal cells composition in the TA and ESM patches. CD31, alpha smooth muscle actin- (α-SMA), CD3-, and CD68-positive cells in the neointima in both groups were observed (Figure 3A). Immunofluorescence showed that there was a layer of von Willebrand factor (vWF)-positive cells on the luminal side, with several layers of α-SMA-positive cells in the neointima in both patches (Figure 3B). Similar re-endothelialization of the neointima was observed in both the TA and ESM patches; compared to the TA patch, the α-SMA-positive smooth muscle cells were not aligned evenly in the ESM patch (Figure 3C). CD34- and nestin-positive progenitor cells also participated in the neointimal formation, and there were a similar number of CD34-positive cells on the luminal side in the neointima of the TA and ESM patches (Figures 3B,C). We then examined the neointimal endothelial cell identity; Ephrin-B2 and dll-4 are arterial endothelial cell markers (Bai et al., 2018b). In both TA and ESM patch groups, the endothelial cells expressed Ephrin-B2 and dll-4 markers, and there was also a similar number of Ephrin-B2- and dll-4-positive cells in the neointima (Figure 3C). These results mean that the neointimal endothelial cells acquire arterial identity in the arterial microenvironment (Figure 3).

**FIGURE 4:**
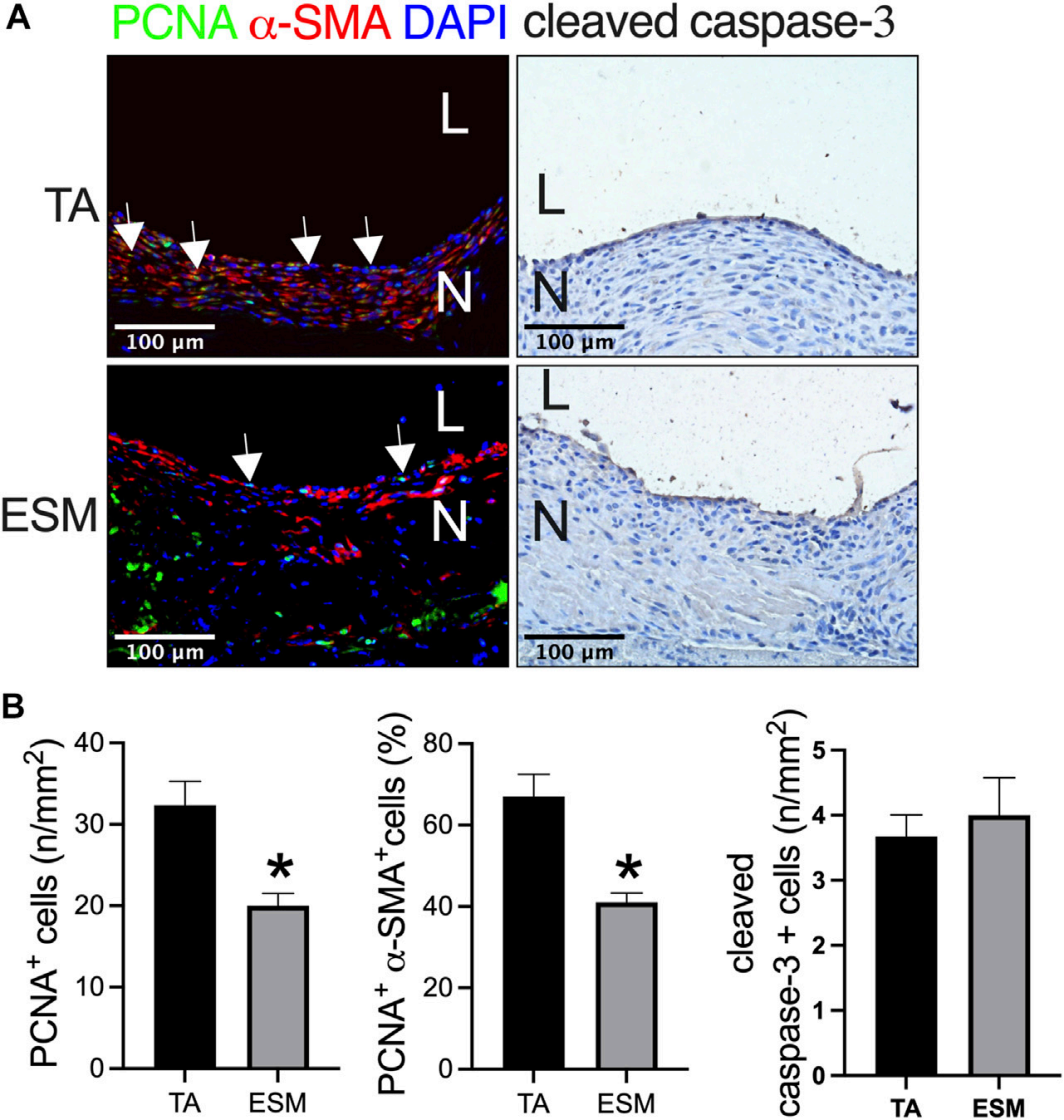
Neointimal cell turnover after TA and ESM patch angioplasty. **(A)** Photograph of immunofluorescence staining for PCNA (green), α-actin(red), and DAPI (blue); photograph of immunohistochemistry staining for cleaved caspase-3; scale bar, 100 μm; L, lumen; N, neointima; white arrows show the positive cell, *n* = 3. **(B)** Bar graphs showing the neointimal PCNA-positive cell numbers (*p = 0.0208, *t*-test), PCNA and α-actin dual-positive percentage (*p = 0.0121, *t*-test), and cleaved caspase-3-positive cell numbers (*p = 0.6433, *t*-test) after TA and ESM patch angioplasty; *n* = 3.

Cell proliferation plays a role in neointimal hyperplasia. We also examined the cell turnover in the neointima, and there were proliferating cell nuclear antigen (PCNA) and α-SMA dual-positive cells in the neointima in both the groups (Figure 4A). There was a smaller number of PCNA-positive cells, and PCNA and α-SMA dual-positive cells in the ESM patch when compared to the TA patch (Figures 4A,B). There were very few cleaved caspase-3-positive cells in both the groups (Figures 4A,B).

### Heparin-Conjugated Egg Shell Membrane Decreases Neointimal Thickness

**FIGURE 5:**
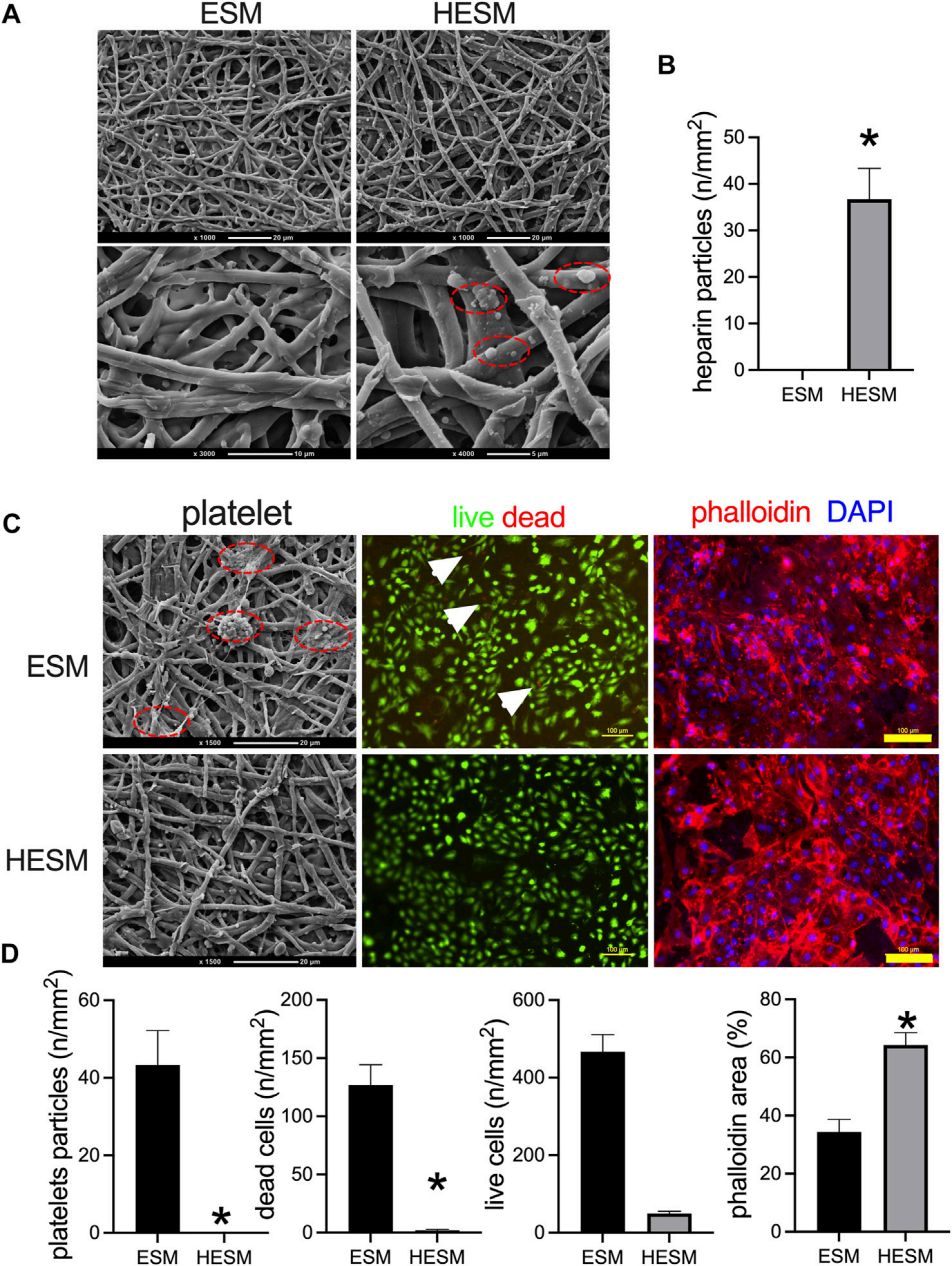
Heparin-coated ESM (HESM) decreases platelets adhesion *in vitro*. **(A)** SEM images of ESM and HESM samples; dashed red line circle showing the heparin particles coated on the HESM patch, *n* = 3. **(B)** Bar graph showing the numbers of heparin particles on the surface of the HESM patch, *p = 0.0053, *t*-test, *n* = 3. **(C)** SEM images of platelet adhesion on the surface of the ESM patch but not the HESM patch; red dashed line circle showing the platelet particles. Fluorescence images of HUVECs cultured on ESM and HESM for 7 days, live (green) and dead (red) cells, white arrows show the dead cells; immunofluorescence showing the nucleus (blue) and phalloidin (red) of cells; *n* = 3. **(D)** Bar graph showing the number of platelet particles (*p = 0.0080, *t*-test), dead cell numbers (*p = 0.0051, *t*-test), live cell numbers (*p* = 0.6880, *t*-test), and phalloidin area (*p = 0.0078, *t*-test) on the surface of the ESM and HESM patches; *n* = 3.

A coating of heparin to the biomaterials is commonly used; we have shown heparin coating in our previous researches (Bai et al., 2020; Yan et al., 2020; Wei et al., 2022). We then explored the influence of heparin conjugation to the ESM, and the ESM and HESM were examined by SEM; since heparin is a complex polysaccharide, a specific marker is not always used to detect it. There were heparin particles conjugated to the surface of the EMS (Figures 5A,B). The endothelial cells on the luminal side of the vessel also have an anticoagulation function, and these were always formed several days later, after the formation of neointima, when a prosthetic vascular graft is implanted in the animal's body (Bai et al., 2017a; Bai et al., 2020). Neointimal endothelial cells and heparin play the same role in preventing thrombus formation, so we only tested the platelet accumulation in this experiment. The platelets were found on the ESM surface, while no platelets were found on the HESM (Figures 5C,D). The HUVECs were cultured on the ESM and HESM patches. Fluorescence images of the HUVECs cultured on the ESM and HESM for 7 days showed fewer dead cells in the HESM patch (Figures 5C,D); there were also more cells on the HESM patch at day 7 (Figures 5C,D).

**FIGURE 6:**
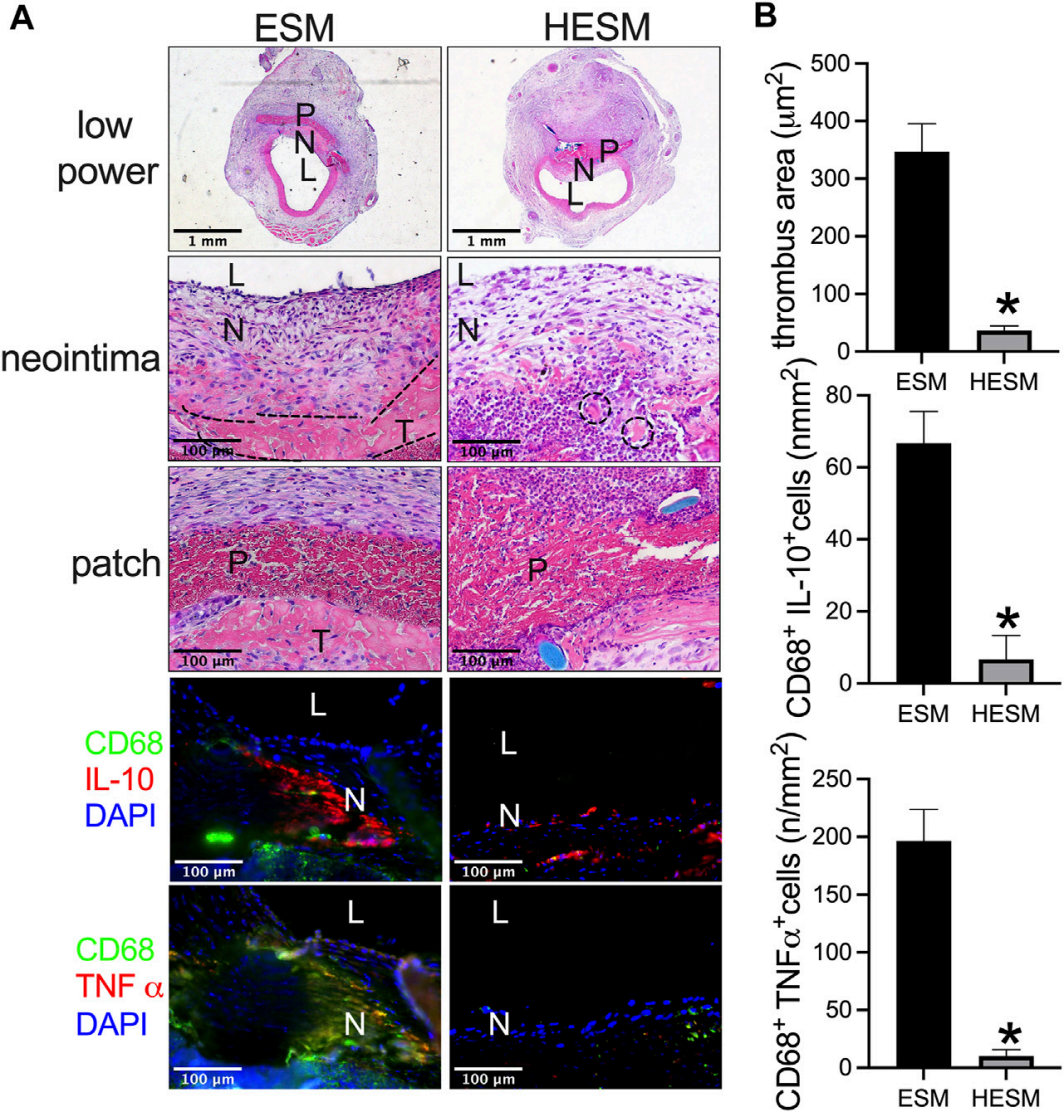
Heparin-coated ESM (HESM) decreases mural thrombus formation and macrophage infiltration after rat aortic patch angioplasty at day 14. **(A)** Photographs of hematoxylin & eosin (H&E) staining of the ESM and HESM patches after angioplasty at day 14, scale bar, 1 mm or 100 μm; L, lumen; P, patch; N, neointima; T, thrombus; dashed line area shows the demarcation of the thrombus. Photograph of the immunofluorescence staining for IL-10 (red), CD68 (green), and DAPI (blue); CD68 (green) and TNF-α (red) in the neointima of the ESM and HESM patches harvested at day 14; scale bar, 100 μm; L, lumen; N, neointima; *n* = 3. **(B)** Bar graph showing thrombus area (*p = 0.0034, *t*-test), CD68 and IL-10 dual-positive cell numbers (*p = 0.0056, *t*-test), CD68 and TNF-α dual-positive cell numbers (*p = 0.0026, *t*-test) in the neointima of ESM and HESM patches harvested at day 14; *n* = 3.

The ESM and HESM were also implanted in the aorta. There was a smaller mural thrombus area in the HESM patch when compared to the ESM patch at day 14 (Figures 6A,B); cells can also migrate into the HESM patch (Figure 6A). We also examined M1 and M2 macrophages in the neointima; there were fewer macrophages, fewer IL-10, and CD68 dual-positive cells (M1), and fewer CD68 and TNF-α dual-positive cells (M2) in the HESM neointima than in the ESM neointima (Figures 6A,B).

## Discussion

In this study, we showed that the ESM can be used as an arterial vascular patch and shared a similar healing process like decellularized TA patch in the rat aortic patch angioplasty model, heparin can be conjugated to the ESM patch (HESM) and showed a smaller mural thrombus area when compared to the control ESM patch; these results showed the potential clinical application of the ESM in vascular surgery.

The ESM supplies a proper environment during the hatch process and is usually considered as waste. But the ESM has a highly pure microfibrous network like the artificial extracellular matrix scaffold and can be used for drug delivery, tissue scaffolds. One group used graphene-layered ESM (GEM) scaffolds that showed better mechanical and hydrophilic properties than those of a raw ESM. The GEM scaffolds can control the adhesion properties of stem cells, enhancing the proliferation and osteogenic properties of the cells compared to the effects of a raw ESM (Park et al., 2019). We previously developed a small-diameter, double-layered ESM/thermoplastic polyurethane (ESM/TPU) vascular graft with a wavy structure, which has the potential to yield rapid re-endothelialization *in vitro*; heparin modification to the ESM surface improved its anticoagulation properties (Yan et al., 2020).

Natural scaffolds or biomimetic materials have shown good results in animal research (Bai et al., 2021b; Xie et al., 2021; Bai et al., 2021c). We have shown that patch angioplasty is a useful model to test new biomaterial scaffold. Decellularized vascular grafts are wildly used in vascular research and show unique merits compared to the prosthetic graft (Niklason and Lawson, 2020), so we compared the decellularized TA patch and ESM patch. There was a similar neointima formed in both the TA and ESM patch groups; CD34 and nestin progenitor cells both participated in the neointimal formation process. These results showed that the ESM patch shared a similar healing process like the decellularized TA patch. Since the ESM showed these excellent results in this model, the ESM may be a novel biomaterial to use in vascular surgery.

Heparin is a classical drug to inhibit acute thrombus formation after vascular interventions both in clinical and basic research. We previously showed that heparin-coated human great saphenous vein patch can decrease neointimal thickness in both the aortic and IVC angioplasty models in rats (Bai et al., 2020), and patch materials can influence the neointimal thickness (Bai et al., 2021d). We further tested the ESM when it was coated with heparin, and both the *in vitro* and *in vivo* experiments data showed that the coated heparin can effectively decrease platelet accumulation *in vitro* and acute thrombus formation *in vivo*. These data show the potential application of the ESM not only as a vascular graft but also a drug delivery system. The HESM had higher cell viabilities than the original ESM because heparin was shown to promote endothelial cell growth by facilitating their interactions with cell surface receptors (Yan et al., 2020).

There are also some limitations in our research. Firstly, we only used small animals, therefore whether the ESM can sustain the blood pressure in large animals should be explored; secondly, the observation time was 2 weeks, therefore a longer time of observation should be tested; thirdly, the application of the ESM on the venous system should also be tried; fourthly, the combination of the ESM with other materials to enhance its mechanical performance requires more experiments.

## Conclusion

By using a rat aortic patch angioplasty model, we showed that the ESM can be used as a vascular patch to repair the rat aorta. It can also be conjugated with heparin to decrease thrombus formation both *in vivo* and *in vitro*. Therefore, we concluded that the ESM may have a potential application in vascular biomaterial research in vascular surgery.

## Data Availability

The original contributions presented in the study are included in the article/Supplementary Material, and further inquiries can be directed to the corresponding authors.
